# Sonographic Diagnosis and Management With Delayed Hysterectomy of Two Cesarean Scar Pregnancies That Developed Into Placenta Percreta: Two Case Reports

**DOI:** 10.7759/cureus.37130

**Published:** 2023-04-04

**Authors:** Katherine T Huebner, Eneka Lamb, Alexandria Weymon, Leigh Seamon, Mili Thakur, Emma Giuliani, Michael Ryan, Marcos Córdoba

**Affiliations:** 1 Maternal Fetal Medicine, Michigan State University College of Human Medicine, Grand Rapids, USA; 2 Gynecologic Oncology, Spectrum Health Medical Group, Grand Rapids, USA; 3 Reproductive Endocrinology and Infertility, The Fertility Center, Grand Rapids, USA; 4 Reproductive, Endocrinology and Infertility, The Fertility Center, Grand Rapids, USA; 5 Radiology, Advanced Radiology Services, Grand Rapids, USA; 6 Maternal Fetal Medicine, Spectrum Health Medical Group, Grand Rapids, USA

**Keywords:** placenta percreta, placenta accreta spectrum disorder, etoposide, delayed hysterectomy, cesarean scar pregnancy

## Abstract

Approximately two-thirds of the patients with a cesarean scar pregnancy (CSP) will develop placenta accreta spectrum (PAS). PAS occurs when the placenta attaches too deeply to the uterine wall, and sometimes, the placenta can extend beyond the uterus, invading surrounding organs. PAS is commonly managed with a cesarean hysterectomy, and these deliveries are often complicated by maternal and fetal morbidity and mortality. However, delaying hysterectomy and using chemotherapeutic agents may be a safe and beneficial alternative. We describe the case of a 32 -year-old G3P2002 with a history of two prior cesarean sections (CS) who was referred to our Maternal Fetal Medicine department due to the concern of a gestational sac embedded in the anterior uterine wall in the cesarean scar. Magnetic resonance imaging (MRI) findings at 33 weeks confirmed that the patient had developed placenta percreta extending into the sigmoid colon. We also describe the case of a 30-year-old G6P4104 with a history of four prior CS who was referred to our department for concern of a pregnancy complicated by CSP. This patient had an MRI performed at 23 weeks that showed placenta percreta invading the bladder. Patients one and two were managed with a staged procedure, with CS followed by a delayed laparoscopic and abdominal hysterectomy, respectively, to minimize bowel and bladder injury. After the CS, the patients subsequently received a five-day course of intravenous (IV) etoposide 100mg/m2, and at six weeks postpartum, the patients had a hysterectomy, both showing resolution of the placenta invasion into the surrounding organs on postpartum MRI and confirmed by tissue pathology reports. Our cases present the challenge in diagnosis and management of the most severe presentation of PAS that varies from the generally accepted management recommendations. Delayed hysterectomy with chemotherapy can be a reasonable, conservative surgical approach in the most severe types of PAS. As in our cases, this management could improve maternal and fetal morbidity and mortality.

## Introduction

Cesarean scar pregnancy (CSP) results in the implantation of the gestational sac into the fibrous tissue scar of a previous cesarean section (CS) [1]. The diagnosis of CSP is based upon the ultrasound (US) detection of an early gestation in the proximity of the hysterotomy of a prior CS in an empty uterine cavity and abundant blood flow around the gestational sac [2]. CSP is estimated to occur between 1:1800 to 1:2656 in all pregnancies [1]. Several management options have been reported in the literature; however, given the rarity of this condition, there is a lack of universally agreed upon and adopted treatment protocols [1-3]. Patients must be counseled on the complications of CSP, such as severe bleeding, rupture of the uterus, fetal demise, or preterm delivery, and as a result, many choose to terminate the pregnancy [3,4]. 

Those who elect to continue with the pregnancy are at risk of developing placenta accreta spectrum (PAS). According to a systemic review, two-thirds of women with a CSP went on to develop PAS [2]. PAS occurs when the placenta attaches too deeply to the uterine wall, and sometimes the placenta can extend beyond the uterus invading surrounding organs such as the bowel or the bladder [5]. In the literature, the incidence of PAS is rising, and recent studies between 2013 and 2015 show that it now accounts for 4.8/10,000 deliveries [5,6]. PAS is commonly managed with a scheduled cesarean hysterectomy between 32 and 34 weeks of gestational age, and these deliveries are often complicated by maternal hemorrhage, urinary tract injury, and admission to the intensive care unit [5]. Due to these severe complications, recent studies in the literature are advocating for conservative management of PAS, which involves performing a CS and leaving the placenta in situ for reabsorption and delaying hysterectomy [5-8]. There is limited literature describing the use and benefits of the addition of chemotherapeutical agents in the management of PAS to help increase placental resorption and decrease maternal morbidity and mortality. Our cases describe the favorable outcome of two patients with CSP who developed a placenta percreta, one of them invading the sigmoid colon and the other the bladder, which was managed with chemotherapy and delayed hysterectomy. The first case of this manuscript was previously presented as an oral presentation at the 2022 AIUM Annual Meeting on March 13, 2022.

## Case presentation

Case 1

**Figure 1:**
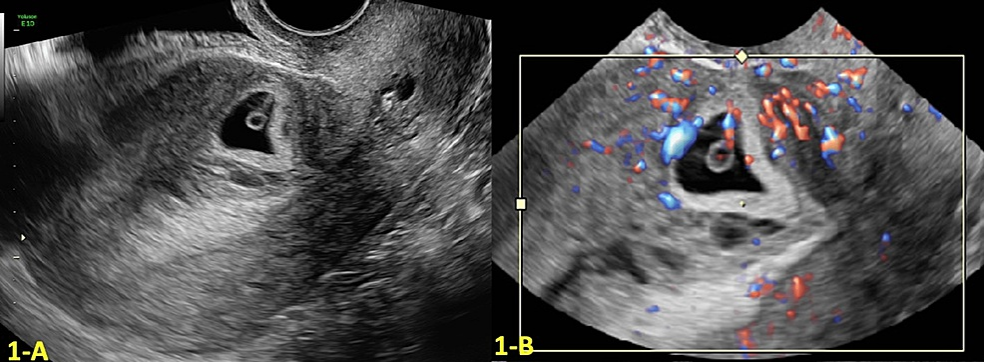
Transvaginal ultrasound (TVUS) findings at six weeks and three days gestational age (GA) A: Transvaginal ultrasound (TVUS) at six weeks and three days gestational age (GA) showing a low-lying gestational sac at the location of the cesarean scar (CS) scar with an empty uterine fundus and cervical canal. B: Color Doppler at six weeks and three days GA showing marked peritrophoblastic flow around the gestational sac consistent with cesarean scar pregnancy (CSP).

A 32-year-old G3P2002 with a history of two prior CS was referred to our Maternal Fetal Medicine department due to the concern of a gestational sac embedded in the anterior uterine wall at the level of the prior cesarean scar. At six weeks of gestational age (GA), a transvaginal ultrasound (TVUS) showed a low-lying gestational sac implanted at the location of the CS scar (shown in Figure 1A). The myometrium over the defect measured 4 mm, and color Doppler US showed a marked peri trophoblastic flow around the gestational sac, consistent with CSP (shown in Figure 1B). Management discussion involved different treatment options, including interruption of the current pregnancy. After extensive counseling, the patient desired to continue with the pregnancy.

**Figure 2:**
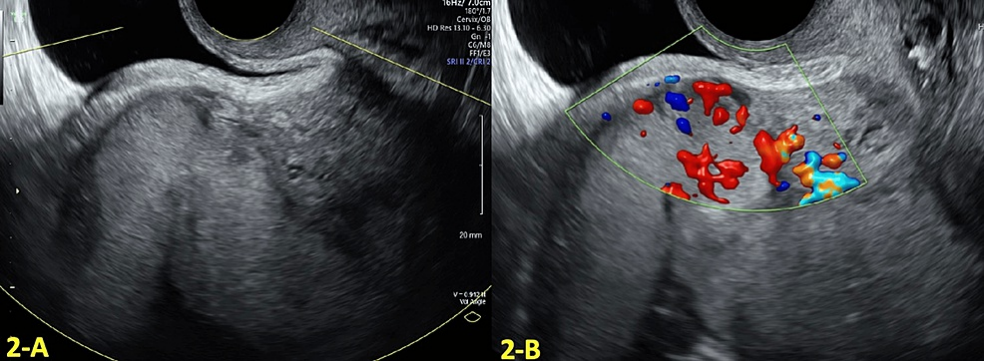
Transvaginal ultrasound (TVUS) findings at 18 weeks and six days gestational age (GA) A: Transvaginal ultrasound (TVUS) at 18 weeks and six days gestational age (GA) showing loss of the hypoechoic boundary of the myometrium in the lower uterine segment and placental bulge. B, Color Doppler at 18 weeks and six days GA showing increased vascularity of the uterine serosa-bladder interface.

US at 18 weeks revealed an anterior grade II placenta previa. There was an area of loss of the hypoechoic boundary of the myometrium in the lower uterine segment (shown in Figure 2A). Color Doppler showed increased vascularity of the uterine serosa-bladder interface (shown in Figure 2B), raising suspicion for PAS.

**Figure 3:**
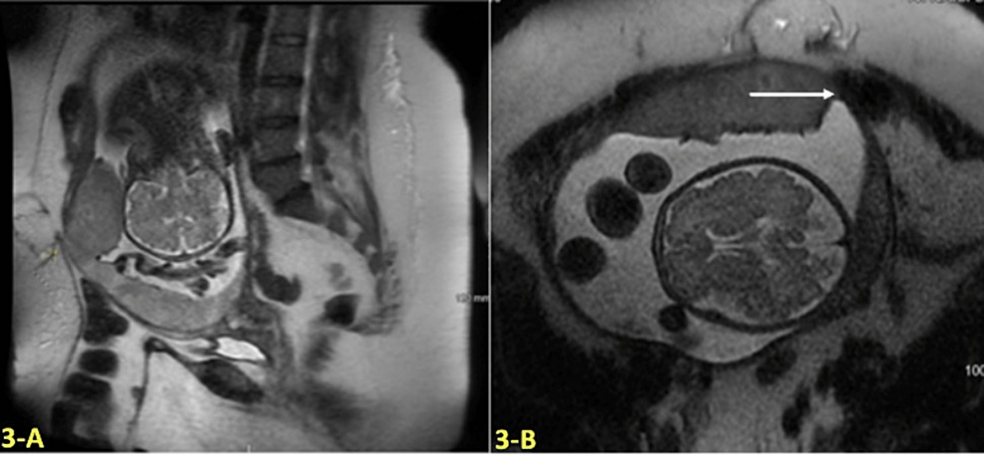
Sagittal single-shot fast spin echo (SSFSE) (A) and Axial T2 weighted SSFSE (B) Magnetic resonance imaging (MRI) findings at 33 weeks gestational age (GA) A and B: Magnetic resonance imaging (MRI) at 33 weeks gestational age (GA) showing a focal bulge of the uterus now in contact with the abdominal wall and a loop of the colon with loss of its normal fat plane indicated by the white arrow.

MRI at 33 weeks confirmed these US findings with the addition of a 5-6 mm focal bulge of the uterus now in contact with the abdominal wall and a loop of the colon with loss of its normal fat plane, suggesting placental invasion into the sigmoid colon as shown in Figures 3A, 3B.

At 34 weeks, after weighing the risks/benefits of different treatment options, our multidisciplinary team (Maternal Fetal Medicine, Gynecologic Oncology, General Surgery, Interventional Radiology, and Urology) considered a staged procedure, with CS followed by a delayed hysterectomy to minimize bowel injury. Bedside US was used to evaluate the placental position, and fundal hysterotomy was made away from the superior edge of the placenta and extended superiorly. The neonate was delivered in the breech position without difficulty. The umbilical cord, membranes, and placenta remained in situ, and the hysterotomy was closed. The area was not further explored because of the risk of many severe potential complications like intraoperative hemorrhage and bowel injury. The patient and neonate did well postoperatively, and she was discharged home on postoperative day five. She reported no post-operative bowel complications. Maternal blood loss was qualified as 1500mL, and the patient was given one unit (350mL) of packed red blood cells (pRBCs), one unit (250mL) of fresh frozen plasma (FFP), and one unit (300mL) of platelets.

The patient had a bilateral uterine artery embolization with particle and micro coil material following her CS and subsequently received a five-day course of etoposide 100mg/m2 IV. The patient tolerated the etoposide well and reported mild alopecia.

**Figure 4:**
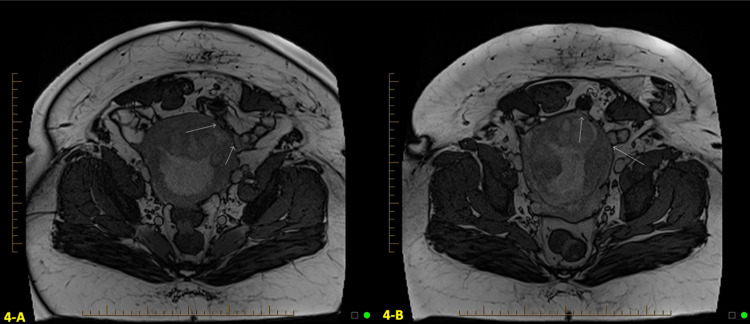
Axial fast imaging with steady-state free precession (FISP) Magnetic resonance imaging (MRI) findings at four weeks postpartum A and B: Magnetic resonance imaging (MRI) at four weeks postpartum demonstrating an anterior placenta with no evidence of gross extension of the placental tissue through the full thickness of the myometrium. The arrows point at a loop of the small bowel adjacent to the anterior left aspect of the uterine body near the area of the placental wall thickening but do not show any definitive evidence of placental soft tissue extension through the uterine wall to involve this loop of the small bowel.

Follow-up MRI at four weeks post-op showed anterior placental location with complete placenta previa (shown in Figures 4A, 4B). At this time, there was no evidence of gross extension of placental tissue through the entire thickness of the myometrium or adjacent parametrial tissue. There was a loop of small bowel adjacent to the anterior left aspect of the uterine body near the area of placental wall thickening, but no definitive evidence of placental soft tissue extension through the uterine wall to involve this loop of the small bowel.

Robotic-assisted laparoscopic hysterectomy was scheduled and performed at six weeks postpartum. Two days before the planned procedure, the patient presented to the emergency department with heavy vaginal bleeding and abdominal pain. A CT angiogram showed no active arterial bleeding. The patient had an uncomplicated robotic multisite hysterectomy with bilateral salpingectomy and robotic ventral incisional hernia repair with Phasix ST mesh and lysis of adhesions surrounding the colon and between the uterus and mesentery. Her estimated blood loss was 100mL, and she was discharged on postoperative day one. Uterine tissue pathology confirmed PAS. There were no immediate postoperative complications.

Case 2

**Figure 5:**
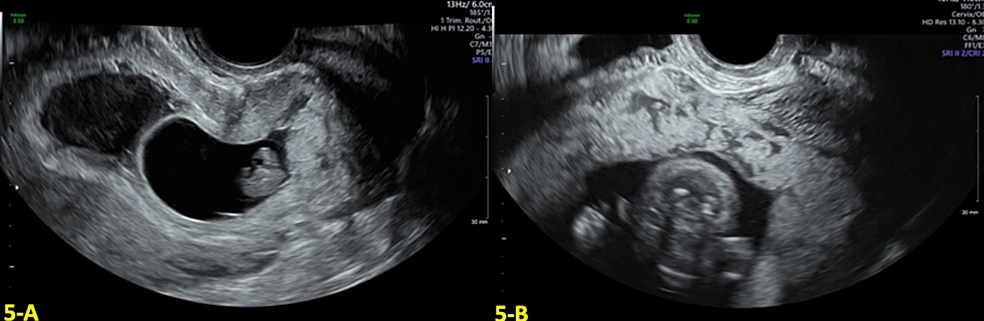
Transvaginal ultrasound (TVUS) findings in the first and second trimesters A: Transvaginal ultrasound (TVUS) at eight weeks and five days gestational age (GA) showed a low-lying gestational sac at the location of the cesarean section (CS) scar. B: Anterior placenta previa with multiple placental lakes at 16 weeks and four days GA.

A 30-year-old G6P4104 with a history of four prior CS was referred to our department at eight weeks gestation for concern of a pregnancy complicated by CSP. At eight weeks GA, TVUS revealed a low-lying gestational sac implanted near the location of the CS scar with a thin myometrium over the cesarean scar defect (shown in Figure 5A). Color Doppler US demonstrated marked peri trophoblastic flow around the gestational sac, which can be suggestive of CSP. The patient was counseled on the risks of CSP but elected to continue with expectant management of the CSP and to continue with the pregnancy.

US at 16 weeks showed an anterior placenta previa with multiple placental lakes and increased vascularity in the lower uterine segment, as shown in Figure 5B. These findings were highly suggestive of placenta accreta. 

**Figure 6:**
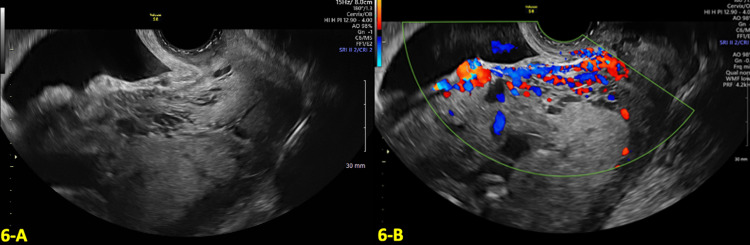
Transvaginal ultrasound (TVUS) findings at 23 weeks and one day gestational age (GA) A: Anterior placenta previa with placenta lacunae and an irregular bladder line highly suspicious for percreta at 23 weeks and one day gestational age (GA). B: Increased vascularity on Color Doppler transvaginal ultrasound (TVUS) at the uterine-serosa bladder interface at 23 weeks and one day GA.

**Figure 7:**
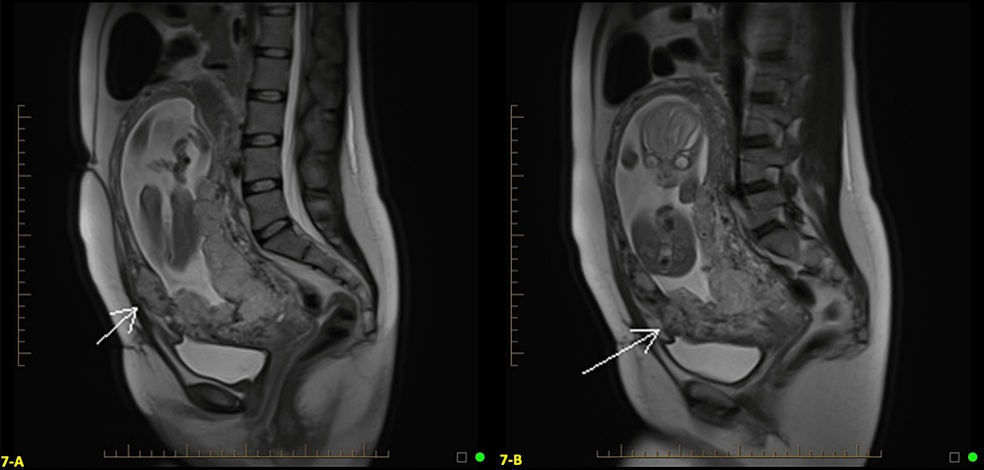
Sagittal half Fourier acquisition single-shot turbo spin echo (HASTE) Magnetic resonance imaging (MRI) findings at 23 weeks and one day gestational age (GA) A and B: Magnetic resonance imaging (MRI) at 23 weeks and one day gestational age (GA) confirming placenta previa with findings highly suspicious for placenta percreta. The placenta appears to involve the anterior abdominal wall in the midline, indicated by the white arrow (Image A), and involves the bladder (Image B).

At 23 weeks, the patient was admitted to the hospital for vaginal bleeding with an estimated blood loss of 200mL. She had previously had two other prior episodes of vaginal bleeding, one at 12 weeks and the other at 16 weeks. Neither prior episode required admission and stopped on its own. There were no signs of active hemorrhage, so the decision was made to monitor the patient for any signs of decompensation and administer two doses of 12 mg of betamethasone for fetal maturity in case the active bleeding restarted, and delivery was as indicated. We monitored the patient’s vitals and trended her coagulation studies and CBC every 12 hours, monitoring for signs of active bleeding. To monitor fetal well-being, we placed her on continuous electronic fetal monitoring for signs of fetal distress or preterm labor. US at admission demonstrated an anterior placenta previa with placenta lacunae and an irregular bladder line, which was highly suspicious for placenta percreta (Figures 6A, 6B). An MRI at 23 weeks confirmed placenta previa, with findings highly suspicious for placenta percreta involving the bladder and possibly the anterior abdominal wall in the midline, as shown in Figures 7A, 7B.

The patient remained stable in the hospital for 13 days before vaginal bleeding increased, requiring delivery. At 24 weeks, our multidisciplinary team elected a staged procedure, with CS followed by a delayed hysterectomy and a bilateral uterine artery embolization with embospheres and gel foam to minimize injury to the bladder and abdominal wall. The staged procedures were elected secondary to the degree of placental invasion into her bladder and abdominal wall to decrease the possibility of bowel injury and hemorrhage that could have occurred if her hysterectomy was performed immediately following the CS. The patient's quantitative blood loss was 500mL. Post-operatively, she did well and received a five-day course of etoposide 100mg/m2 IV. The patient only experienced minimal side effects (night sweats and nausea) from the chemotherapy. The neonate spent 114 days in the neonatal intensive care unit (NICU) and was discharged in stable condition.

**Figure 8:**
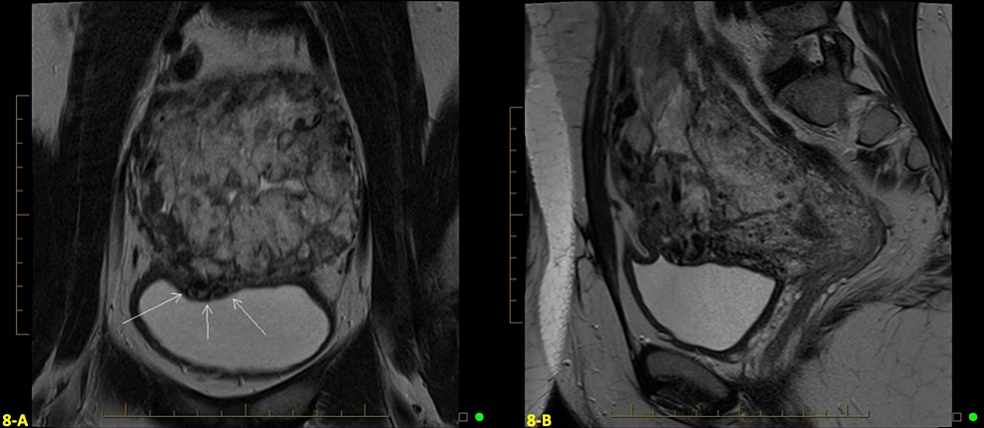
Axial (A) and Sagittal (B) T2 weighted magnetic resonance imaging (MRI) findings at four weeks postpartum A and B: Follow-up magnetic resonance imaging (MRI) at four weeks postpartum revealed a persistent placenta percreta to the anterior dome of the bladder demonstrated by the arrows.

Follow-up MRI at four weeks postpartum revealed a persistent placenta percreta to the anterior dome of the bladder, so the decision was made to perform a total abdominal hysterectomy, bilateral salpingectomy, cystoscopy, and ureteral stent placement at six weeks postpartum to avoid the risk of potential bladder injury (shown in Figures 8A, 8B). The placenta was dissected off the bladder; however, no bladder resection was needed. Uterine tissue pathology confirmed PAS. Her estimated blood loss was 500mL. The patient did well postoperatively and had no further complications. She was discharged on postoperative day two.

## Discussion

Our cases present the challenge in diagnosis and management of the most severe cases of PAS that varies from the generally accepted management recommendations and brings attention to an alternative option of the management. We demonstrated that delayed hysterectomy with chemotherapy can be a reasonable, conservative surgical approach in the most severe types of placenta percreta. 

In the literature, it has been documented that delaying hysterectomy gives time to reduce placental vascularity and its involvement in surrounding organs, avoiding significant maternal hemorrhage and the need for blood transfusions [7]. In 2022, Sentilhes et al. published a prospective study of women with PAS comparing maternal outcomes after cesarean hysterectomy versus leaving the placenta in situ during cesarean birth and found that conservative management was associated with lower incidences of women requiring more than four units packed red blood cells (pRBCs) within six months of delivery [5]. A prior French retrospective analysis demonstrated 78% of women (n=167) were successfully treated with conservative management avoiding a hysterectomy altogether, and 6% of those treated conservatively had severe maternal morbidity defined as sepsis, septic shock, peritonitis, uterine necrosis, postpartum uterine rupture, fistula, injury to adjacent organs, acute pulmonary edema, acute renal failure, deep vein thrombophlebitis or pulmonary embolism or maternal death [8]. The route of hysterectomy should be based on postpartum US and MRI findings. In patients with adequate placental resorption and no further invasion into surrounding tissues, a laparoscopic hysterectomy can be considered as in the first case presented. However, in patients where the placenta remains invasive, an abdominal hysterectomy should be considered to avoid further injuring the surrounding structures and requiring more extensive surgery. In our patients, delaying hysterectomy prevented any serious morbidity and mortality. Patient one only required one unit of pRBCs, and her total blood loss estimate for both procedures was 1600mL; patient two did not receive any transfusions and had a total estimated blood loss of 1200mL, including prior vaginal bleeding.

The use of chemotherapeutical agents in patients with PAS to help increase placental reabsorption has been included in a few studies. A systematic review found no significant difference in patients with PAS treated with chemotherapy; however, only a small portion of the patients were treated with chemotherapy (30 of 72) [9]. In the literature, most studies include methotrexate, and few mention other agents, such as etoposide. Methotrexate has been documented to cause one maternal death during the conservative management of a patient with PAS due to myelosuppression and nephrotoxicity [10]. We believe that receiving a five-day course of etoposide 100mg/m2 IV could be a superior agent to methotrexate in the management of PAS. Etoposide halts cell division by creating and accumulating breaks in deoxyribonucleic acid (DNA) which leads to cell death [11]. When used in cases of morbidly adherent placenta, it causes cell death and necrosis, leading to decreased placental vascularity and increased resorption of the placental tissue allowing for better surgical dissection [11]. Etoposide has been described in the treatment of ectopic pregnancy and trophoblastic placental tumors showing good efficacy [11,12]. Adverse effects of etoposide include alopecia, neutropenia, and the risk of secondary malignancy (leukemia, breast and colon cancer and melanoma) [12,13]. Most of the side effects documented are based on treatment protocols that require more cycles of chemotherapy. Our patients only required a single five-day course of etoposide, decreasing their risks of secondary malignancy. Future studies should further investigate its benefits, dosing, and mode of administration as it showed promising results in our patients and allowed them to avoid the need for bowel or bladder resection.

When deciding whether a patient is a good candidate for our proposed stagged procedure with adjunctive chemotherapy versus a cesarean hysterectomy, many factors should be considered. The stagged procedure with chemotherapy should only be offered to patients at tertiary hospitals where a multidisciplinary team is available at all times. Additionally, we propose that it should be recommended for all patients with severe PAS where there is a large concern for potentially serious injury to the surrounding structures and organs during a traditional cesarean hysterectomy at birth.

## Conclusions

In conclusion, delayed hysterectomy with the addition of chemotherapeutic agents gives time for placental reabsorption and can decrease the placenta's involvement in surrounding organs. Based on the favorable outcomes of our two patients, we believe that this protocol should be considered in the most severe cases of PAS to reduce blood loss and the need for bowel or bladder resection. With the incidence of CSP and PAS on the rise, future research should focus on evaluating the effectiveness of delayed hysterectomy and etoposide in cases of PAS extending into surrounding organs. This is important to improve maternal morbidity and mortality of future patients with severe PAS.
